# Health Insurance Literacy and Medical Care Avoidance Among International Students: A Case Study

**DOI:** 10.3389/ijph.2023.1605788

**Published:** 2023-10-06

**Authors:** Echu Liu, Samantha A. Arledge, Miao Cai, Donghua Tao, Wei Li

**Affiliations:** ^1^ Department of Health Management and Policy, Saint Louis University, Saint Louis, MO, United States; ^2^ Department of Epidemiology, School of Public Health, Sun Yat-Sen University, Guangzhou, China; ^3^ Medical Libary, Saint Louis University, Saint Louis, MO, United States; ^4^ Department of Data Science, Dongfang College, Zhejiang University of Finance and Economics, Haining, China

**Keywords:** health insurance literacy, health care avoidance, international students, United States, case study

## Abstract

**Objectives:** This research examines the health insurance literacy and healthcare utilization of international students attending a university in the US Midwest.

**Methods:** One hundred and forty-three undergraduate and graduate students attending a midsize metropolitan university in the Midwest completed an online survey in early 2022.

**Results:** Many students surveyed could not identify the definitions of basic terms, such as copay. Furthermore, about 80% of students surveyed could not determine their financial responsibilities in two medical settings. Regression results show that the continent they are from and the length of their stay in the United States significantly predict their understanding of key health insurance terms. More than half of the international students surveyed indicated they often feel confused about their health insurance (57.34%). Only about 20% have delayed or skipped care due to unfamiliarity with the health insurance system.

**Conclusion:** The health insurance literacy of most international students at the midwestern university we surveyed is not ideal. This and possibly other universities in the United States should take more initiatives to help their international students understand the health insurance system.

## Introduction

According to several national surveys, many Americans are health insurance illiterate [1–8], meaning that they lack “the capability to find and evaluate information about health plans, select the best plan given financial and health circumstances, and use the plan once enrolled” [9]. For example, the American Institute of CPA’s survey showed that more than half of respondents could not correctly identify at least one of the following terms: premium, deductible, and copay [1]. Another survey by the Kaiser Family Foundation in 2014 concluded that many participants seemed to understand the basic terms of their health insurance. Still, most could not correctly calculate their financial responsibility in a medical scenario [3]. A more recent survey by Forbes showed that more than half of participants could not answer the definitions of basic but critical health insurance terms, such as co-insurance [8]. Several scholarly articles also echo the findings from these survey studies [10–13]. Past studies also found that health insurance literacy differs by age, care use, ethnicity, education, health status, income, insurance status, languages spoken, health behaviors, health system characteristics, marital status, and race [3, 4, 8, 10, 11, 13–17].

Given the complexity of the US healthcare system, lacking health insurance literacy can negatively impact an individual’s ability to make informed choices when seeking medical care and, consequently, harm personal health and financial well-being—findings from the literature support this statement. For example, past studies found low health insurance literacy is often associated with the delay or avoidance of care or treatment [6, 18, 19], lower confidence in getting care when needed [20], lacking a usual source of care [20], problems with paying medical bills [20], unnecessary medical spending [21, 22], and worse health outcomes [15].

Many students from other countries attend colleges in the United States every year. Although they account for only a tiny percentage of the US college student population [23], their contribution to the US economy is significant. For example, a recent report showed that international students contributed more than $28 billion to the US economy and supported more than 300,000 jobs during the 2020–2021 academic year [24]. Given this notable contribution, most universities provide various support to help their international students acclimate [25]. However, like any newcomer to the United States, adapting to a new system is challenging for international students because of potential language barriers, the difference in communication styles, and the long process of adjustment [26], although they have support from their universities.

Healthcare is one central area to which international students need to adjust [27–29]. Many international students do not adapt well to the US healthcare system because, as some literature reports, they generally underutilize their medical care [27, 30]. Many international students rely on the internet for self-diagnosis and self-treatment with the medicine they bring from their home countries [27, 28]. This problematic behavior is related to potential adverse outcomes, such as disease progression and contagious illnesses, negatively impacting many international students’ health and well-being.

The underlying reasons for international students’ resistance to healthcare use are various because of their diverse cultural backgrounds [27]. One study suggested that one common cause is the difficulty in understanding the US healthcare system, which is significantly different from those in their home countries [27]. According to the same study, insurance terms and terminology, which even many Americans may not understand well, often hinder international students from understanding what services their insurance covers and where they can obtain healthcare [27]. Understanding health insurance is essential to accessing medical care, a critical determinant of quality of life in the United States [31]; thus, international students’ health insurance literacy needs to be studied to ensure their academic success.

Some past qualitative research has examined the health insurance literacy of international students and reported a high prevalence of health insurance illiteracy among international students [27–30]. Furthermore, these qualitative studies show that international students often feel confused and frustrated about the US health insurance system [28] and their lack of understanding [29]. However, quantitatively, few studies has examined international students’ health insurance literacy. This study attempts to fill this gap in the literature by surveying international students at a university in a metropolitan Midwestern area, guided by the following four questions: (a) How do international students perform on an instrument for measuring health insurance literacy? (b) What are the characteristics associated with international students’ health insurance literacy level? (c) What is the prevalence of medical care avoidance due to a lack of understanding health insurance among international students? (d) What are international students’ concerns when delaying or avoiding medical care due to insufficient knowledge of health insurance?

## Methods

### Setting

About 700 international students were enrolled at the institution when this study was conducted. The institution offers baccalaureate, master’s, and doctoral degrees. The campus is in the center of a metropolitan area in the Midwest. An office of international services on campus is available to help students and scholars from other countries settle after they arrive. Before starting, this study was approved by this university’s institutional review board (IRB).

### Procedures

The office of international services sent a link to our survey on the Qualtrics platform to all international students on campus on 4 February 2022. The survey had two parts. The first part was an IRB-approved informed consent. Students had to sign the informed consent electronically before responding to the online questionnaire. The second part of the survey was the questionnaire for this study’s data collection. Between 4 February 2022, and 29 March 2022, 241 students consented to participate in the study, but only 157 finished the survey. Students who completed the survey received a $5 Amazon gift card, and their responses were recorded anonymously.

Before distributing the survey to all international students on this institution’s campus, 8 alumni reviewed the questionnaire in a focus group setting and provided feedback. Their comments were considered when revising the questionnaire for distribution.

### Questionnaire

The questionnaire, adapted from one by Nobels et al. (2019) [32], consisted of four parts. The first part included questions about the demographic characteristics of this study’s potential participants. These questions asked about their current standing at the university, the college they attended, their age, their gender, the continent they are from, the type of health insurance they had, the payer of their health insurance, health status, income, and the length of their stay in the United States. The second part measured the health insurance literacy of potential study participants. The third part solicited potential participants’ self-perceived understanding of health insurance terms before and after answering the 17 questions in the second part of the questionnaire. Responses to these two questions were recorded on a 5-point Likert-type scale ranging from 1 = extremely well to 5 = not well at all. The last part had two questions. The first one asked if the study participants ever felt confused about using their health insurance or picking a health insurance plan. The second question asked if a lack of understanding of their health insurance plan stopped them or significantly delayed them from seeking medical care. The responses to these questions were recorded as “yes,” “maybe,” or “no.” If the answer to either of these questions was “yes” or “maybe,” a follow-up question asked for a brief explanation to complete the questionnaire. These responses provided qualitative information that the quantitative portion of the questionnaire could not offer. The questionnaire is available upon request.

### Health Insurance Literacy Instrument

The second part of the questionnaire, the instrument for measuring health insurance literacy, was used to test international students’ understanding of key health insurance terms and the concept of cost-sharing and how to calculate healthcare costs. The first section consisted of 17 questions related to insurance vocabularies probing potential study participants’ recognition of the definitions of the following terms: co-insurance, copay, deductible, out-of-pocket cost, out-of-pocket maximum, premium, brand-name drugs, generic drugs, formulary, annual limit, benefits, network, prior authorization, referral, summary of benefits and coverage, health maintenance organization (HMO), and preferred provider organization (PPO). The second section had two financial responsibility multiple-choice questions asking each respondent to determine how much they must pay for in certain medical scenarios. Qualtrics randomized questions and answer choice order in this instrument for each respondent.

### The Score of Health Insurance Terms

We followed Nobels et al. (2019) [32] to facilitate our analysis to measure international students’ knowledge of key health insurance terms. We first assigned a score of 1 to each question in the instrument if the response was correct and 0 otherwise. Afterward, we calculated each respondent’s raw health insurance vocabulary score by summing the number of correctly answered questions. Then, we assessed everyone’s knowledge of key health insurance terms by dividing the raw score by 17 and multiplying the resulting quotient by 100 to convert it into a score of health insurance terms on a 0–100 scale.

### Analysis

Descriptive statistics were reported for the characteristics of our survey participants. An analysis of variance (ANOVA) and t tests were performed to determine whether there was a statistically significant difference in the mean score for knowledge of health insurance terms by group characteristics. Based on the test results, we generated the independent variables for multiple linear regression to explore the association between the covariates and students’ knowledge of health insurance terms. Spearman’s rank correlation ( $r_{s}$ ) was calculated to determine the strength and direction of the association between our study participants’ self-rated understanding of health insurance terms before answering the questions on key health insurance terms and their scores on these questions. Kendall’s rank correlation ( $r_{k}$ ) was computed to determine how students’ knowledge of health insurance terms related to their performance on the second part of the instrument, financial responsibility, in two medical scenarios. Answers to the free text questions related to respondents’ confusion about health insurance and their experience of delaying or avoiding medical care were analyzed using a thematic approach.

## Results

As mentioned previously, 241 international students consented to participate in the study; however, only 157 completed the survey. Among these 157 responses, Qualtrics found 8 (5.1%) duplicates, 6 (3.82%) likely fraudulent, and a dot. Therefore, 143 answers (91.08%) were available for analysis.

As Table 1 shows, most of our study sample consisted of master’s students (51.75%), and about one-third (37.76%) were students from the College of Arts and Sciences. Additionally, more than half (58.74%) of our sample were females, and most were from Asia (72.73%). Moreover, more than half (58.04%) of our study sample had student health insurance from the university, and about 40% of their families paid the insurance premium for them. Most of our study sample rated their health status as “good” (41.96%), had a monthly disposable income of less than $1,000 (67.83%), and had been in the United States for less than 1 year (50.35%).

**TABLE 1:** Characteristics of respondents ( N = 143). United States, Midwest 4 February 2022.

Characteristics	n (%)	Mean (Standard deviation)
Current standing	11 (7.69)	
Undergraduate-1st year	4 (2.80)	
Undergraduate-2nd year	11 (7.69)	
Undergraduate-3rd year	11 (7.69)	
Undergraduate-4th year or beyond	11 (7.69)	
Graduate: Master’s	74 (51.75)	
Graduate: Doctoral	23 (16.08)	
Graduate-professional: JD, MD, etc.	11 (7.69)	
School/college of enrollment		
Arts and Sciences	54 (37.76)	
Business	16 (11.19)	
Education	2 (1.40)	
Engineering, Aviation, and Technology	23 (16.08)	
Health Sciences	12 (8.39)	
Law	2 (1.40)	
Medicine	12 (8.39)	
Nursing	7 (4.90)	
Professional Studies	5 (3.50)	
Public Health and Social Justice	10 (6.99)	
Age		25.83 (6.02)
Gender		
Female	84 (58.74)	
Male	59 (41.26)	
Continent respondents are from		
Africa	11 (7.69)	
Asia	104 (72.73)	
Europe	11 (7.69)	
North America	7 (4.90)	
South America	10 (6.99)	
Insurance		
Have student health insurance from the university	83 (58.04)	
Other	60 (41.96)	
Payer of premium		
Graduate student subsidy	19 (13.29)	
Scholarship	20 (13.99)	
Self	49 (34.27)	
Family	55 (38.46)	
Self-rated health status		
Poor	4 (2.80)	
Fair	31 (21.68)	
Good	60 (41.96)	
Very good	48 (33.57)	
Approximate monthly disposable income		
Less than $1,000	97 (67.83)	
$1,000–$1,999	31 (21.68)	
$2,000 or above	15 (10.49)	
Length of stay in the United States		
Less than 1 year	72 (50.35)	
1 year to less than 2 years	18 (12.59)	
2 years to less than 3 years	8 (5.59)	
3 years to less than 4 years	16 (11.19)	
4 years to less than 5 years	6 (4.20)	
5 years or more	23 (16.08)	

**FIGURE 1:**
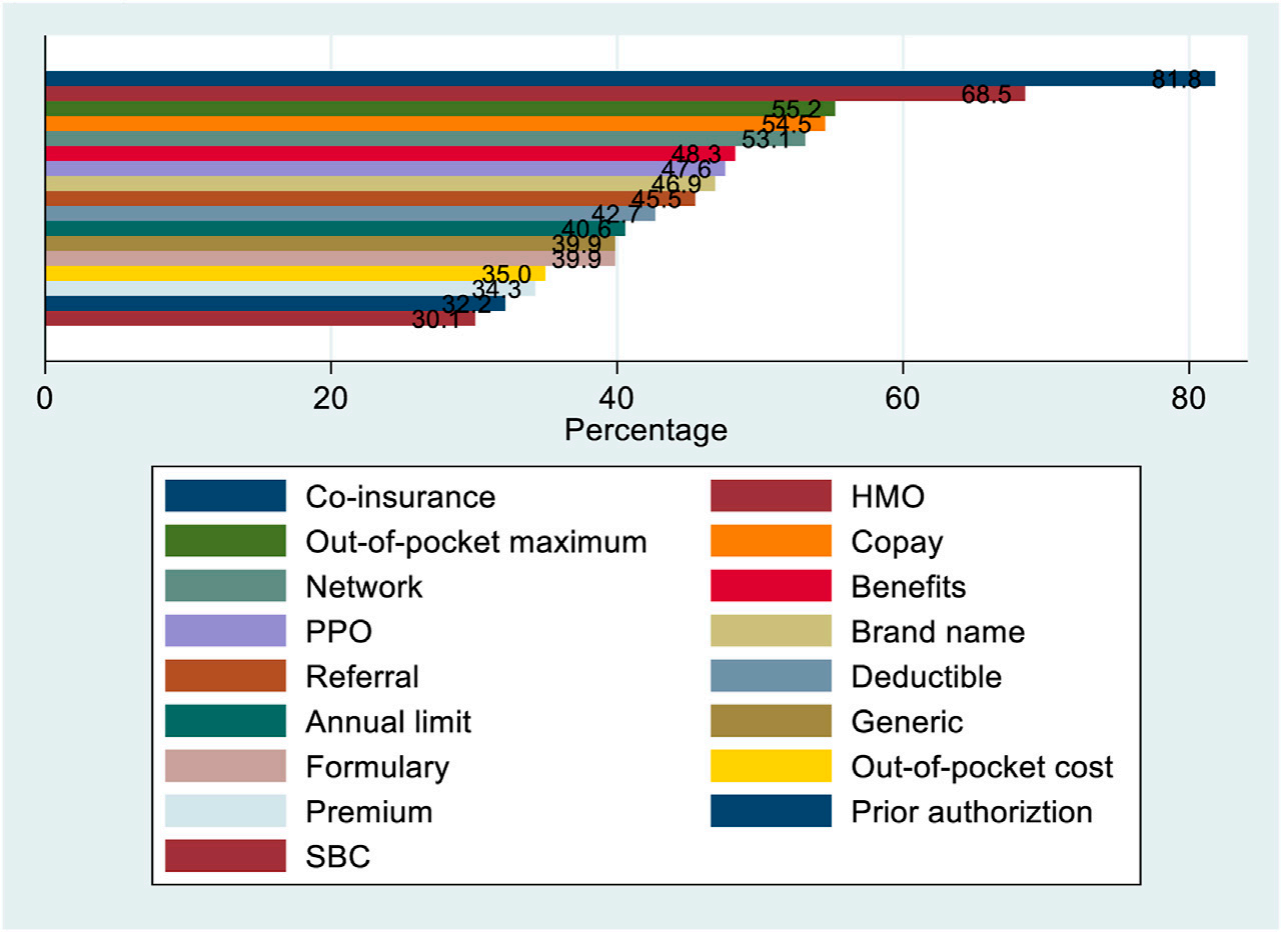
Percentage of respondents who incorrectly identified vital health insurance terms (N = 143). United States, Midwest 4 February 2022.

**FIGURE 2:**
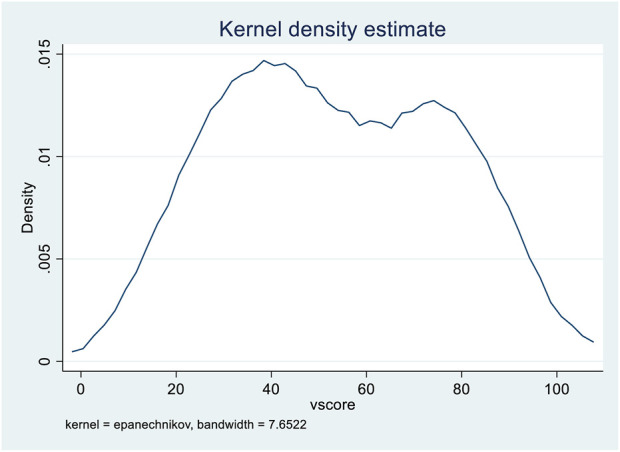
Distribution of health insurance vocabulary score United States, Midwest 4 February 2022.

Figure 1 shows that more than half of our study sample could not define co-insurance, HMO, out-of-pocket maximum, copay, and network. Also, almost 50% of our study sample could not remember the meaning of benefits and PPO. The average score of health insurance vocabulary was 53.18, with a standard deviation of 22.94. The median score was 52.94, slightly lower but almost equal to the mean. The range of this scaled score is vast, with a low score of 5.88 and a high score of 100.00. Figure 2 shows that the health insurance terms score distribution was bimodal, with two peaks around 40 and 80. The $\chi^{2}$ and t statistics reported in Table 2 show that, on average, respondents’ knowledge of health insurance vocabulary differs significantly by their current standing at the university, the continent they are from, the type of health insurance they have, and their length of stay in the United States.

**TABLE 2:** Tests of group differences in scores of health insurance vocabulary knowledge by demographic characteristics ( N = 143). United States, Midwest 4 February 2022.

Categories of characteristics	$\chi^{2}$ ( p value)	t ( p value)
Current standing	2.19 (0.05)	
School/college of enrollment	1.87 (0.06)	
Age		0.78 (0.44)
Gender	1.05 (0.31)	
Continent respondents are from	6.10 (0.00)	
Insurance	6.02 (0.02)	
Payer of premium	0.30 (0.60)	
Self-rated health status	1.20 (0.31)	
Approximate monthly disposable income	1.44 (0.24)	
Length of stay in the United States	2.99 (0.03)	

Table 3 presents the results of the multiple linear regression. Given that our study sample is from a small population, we applied the finite-population correction factor to obtain the standard errors reported in this table [33]. As indicated in the table, undergraduate students were estimated to have lower knowledge of health insurance terms, all else equal. Even so, this effect is statistically insignificant. Doctoral students were reported to have a slightly higher score in health insurance terms, holding other things constant, but this estimated effect is also statistically insignificant. Asian students, according to this table, had a lower score on the knowledge of key health insurance terms by approximately 9 points, ceteris paribus. However, this estimate is statistically insignificant. Students from Europe, North America, and South America reported understanding key health insurance terms better. However, this result is statistically significant only for students from North America ( $\hat{\beta }=20.58,p<0.01$ ). Students with student health insurance from the university were estimated to have higher scores by 2.85 points on average, other things being equal, on health insurance vocabularies, but this effect is statistically insignificant. Based on the coefficient estimates reported in Table 3, overall, the higher the students’ health insurance scores, the longer they had been in the United States. Nevertheless, this effect is substantial and statistically significant only for students who have been staying in the United States for more than 5 years ( $\hat{\beta }=11.90,p<0.05$ ).

**TABLE 3:** The association between characteristics and health insurance vocabulary knowledge score^a^. United States, Midwest 4 February 2022.

Characteristics	Estimate	*SE* ^b^	95% CI^c^		*p*
			*LL* ^d^	*UL* ^d^	
Current standing					
Undergraduate	−1.96	4.40	−10.66	6.74	0.66
Master’s programs (reference)					
Doctoral programs (PhD, MD, DDS, JD, etc.)	1.72	5.05	−8.27	11.71	0.73
Continent the respondents are from					
Africa (reference)					
Asia	−9.27	6.17	−21.48	2.94	0.14
Europe	3.56	8.59	−13.44	20.55	0.68
North America	20.58	6.38	7.96	33.19	0.00
South America	8.19	7.35	−6.34	22.72	0.27
Having student health insurance					
Yes	2.85	3.78	−4.63	10.33	0.45
No (reference)					
Length of stay in the United States					
Less than 1 year (reference)					
1 year to less than 3 years	1.06	4.53	−7.89	10.02	0.81
3 years to less than 5 years	2.76	4.68	−6.50	12.02	0.56
5 years or more	11.90	4.68	2.63	21.16	0.01

^a^ N = 143.

^b^ SE, standard error.

^c^ CI, confidence interval.

^d^ LL, lower limit; UL, upper limit.

For the two questions on financial responsibility, only 21.68% of our study sample answered the first question correctly, and 22.38% answered the second question correctly, as Table 4 shows. Supplementary Table S1 presents the distribution of our study sample’s self-rated understanding of health insurance terms before and after answering the health-insurance-related questions (the 17 multiple-choice questions on key health insurance terms and 2 questions about financial responsibility) in the instrument. As the table demonstrates, the percentage of our study sample who stated that they understood health insurance terms “extremely well,” “very well,” or “moderately well” decreased after respondents had answered the health-insurance-related questions (4.9%–3.5%, 16.78%–8.39%, and 41.96%–27.27%, respectively). In contrast, the percentage of “slightly well” or “not well at all” responses increased after respondents had answered the health-insurance-related questions (24.48%–30.07% and 11.89% to 30.77). Additionally, our study sample’s self-rated understanding of the health insurance terms before answering the questions in the health insurance literacy instrument weakly correlated with their score on health-insurance-related terms ( $r_{s}=-0.10,p>0.05$ ). In other words, those who self-rated their health insurance knowledge higher did not necessarily correctly identify more key health insurance definitions. Moreover, our survey participants’ scores on key health insurance terms also weakly correlated with their ability to compute their financial responsibility in the two medical scenarios ( $r_{k}=-0.02,p>0.05$ ). This result means knowledge of key health insurance terms does not predict the respondents’ ability to determine their shares of medical costs.

**TABLE 4:** Questions, options, and students’ answers for the two financial responsibility questions ( N = 143). United States, Midwest 4 February 2022.

Question	Options	*n* (%)
Your insurance company has negotiated a rate of $11,000 for surgery. You have a deductible of $1,000, a co-insurance of 20%, and an out-of-pocket maximum of $4,000. What amount are you responsible for?	$ 4,000	47 (32.87)
	**$ 3,000**	31 (21.68)
	$ 2,200	43 (30.07)
	$ 1,000	22 (15.38)
Your insurance company has negotiated a rate of $100 for an office visit with your primary care provider. You have already paid your deductible for the year, your copayment is $20, and your co-insurance is 10%. What amount are you responsible for?	$30	39 (27.27)
	**$28**	32 (22.38)
	$20	42 (29.37)
	$10	30 (20.98)

Numbers in boldface are the correct answers.

As Supplementary Figure S1 shows, more than half of our sample (57.34%) reported feeling confused about using or picking a health insurance plan, and about 15% (15.38% reporting “maybe”) might face this challenge. Approximately 73% of this study’s participants reported that they do or may feel confused about their health insurance. Supplementary Figure S2 demonstrates that about one-third (28.67%) of our sample reported that a lack of understanding of health insurance has stopped or delayed them from seeking medical care. Approximately 15.38% said that a lack of knowledge of health insurance might cause a delay or avoidance of seeking medical care. About 44% of our survey sample might delay or avoid seeking medical care because of a deficit in understanding their health insurance.

Supplementary Tables S2, S3 summarize the description of the experience of those who answered “yes” or “maybe” to the following two questions: a) Has there been a time when you were confused about using your health insurance or picking a health insurance plan? and b) Has a lack of understanding of your health insurance plan ever stopped you or significantly delayed you from seeking medical care? Of the 103 responses to the first question, 16 were excluded because they could not be categorized due to unclarity. For example, some students responded by saying, “I do not know what to say,” “I cannot remember,” “It is good,” or “Passport.” Fifteen of the 68 responses to the second question were excluded for the same reason.

As demonstrated in Supplementary Table 2, the following are the top three categories of comments on respondents’ experience of feeling confused about using health insurance or selecting health insurance: being uncertain of benefits and coverage (36.78%), being unsure of the cost of care (24.14%), and being ignorant of the care-seeking process (20.69%). These three categories are also the top categories of comments regarding whether study participants had ever delayed or avoided medical care due to the lack of understanding of their health insurance: being unsure of the cost of care (47.17%), being uncertain of benefits and coverage (33.96%), and being ignorant of the care-seeking process (18.87%). Insufficient knowledge of health insurance terms and the complexity of the US healthcare system are also categories of comments for both experiences.

## Discussion

With approximately one million international students, the United States has hosted the highest number of international students for decades [34]. They came to the US for a quality education and a better future. However, as the literature indicates, international students often must handle challenges in many areas [35]. These challenges may include but are not limited to language barriers, academics, social and cultural differences, discrimination, financial stressors, and mental health concerns [36]. There are no easy solutions to these problems [35], so the institutions that international students attend and the campus community must provide resources to help them succeed, not just academically [37]. Otherwise, international students may feel “disappointed, unfilled, and even exploited” [35] and be put in vulnerable positions [38].

As indicated in past qualitative studies, a lack of understanding of the US healthcare system, particularly health insurance, is a common challenge many international students face [27–30]. This problem should be a severe concern to university administrators because it can cause these students to delay or skip necessary medical care, often associated with an adverse health outcome, such as severe illness or costly medical procedures [27]. Unlike past studies, this work uses a mixed approach to examine health insurance literacy among international students at a Midwestern urban university.

In line with previous research, most international students in this study lack a proper understanding of health insurance, based on their responses to an instrument with 17 qualitative questions of key health insurance terms and 2 quantitative financial responsibility questions. Respondents’ actual understanding of health insurance concepts was lower than their perceived understanding, possibly due to their overconfidence in their knowledge of health insurance. One study in the literature obtained a similar finding [5]. The top five health insurance concepts that a high percentage of this study’s participants did not understand include co-insurance, HMO, out-of-pocket maximum, copay, and network. All these terms relate to the cost-control strategies of private health insurance and are possibly uncommon in other countries.

Our regression indicated that those who stay in the United States longer and those from North America better understand key health insurance terms on average. The association between length of stay and knowledge of key health insurance terms is natural because, in general, the longer international students stay, the more chances they have to become familiar with the healthcare system. Students from North America may have fewer cultural and language barriers because of similar cultural traditions and roots of spoken languages; therefore, understandably, their understanding of health insurance terms is better than students from other continents.

Our survey shows that, although approximately 73% of this study’s participants reported that they do or may feel confused about their health insurance, only about 47% of the respondents said that a lack of knowledge of health insurance had prevented them from seeking medical care whenever necessary. A potential explanation for this nonintuitive result is that about half (50.35%) of our analytical sample had been in the United States for less than a year; therefore, their healthcare experience is limited or nonexistent. Moreover, some international students may seek medical care before understanding their health insurance and financial responsibility after using health insurance for care.

Qualitative comments from those students who have felt confused about their health insurance or picking a health insurance plan show that the top three areas they are often confused about include benefits and coverage, cost of care, and the process of seeking care. These are also the top three reasons our respondents delayed or skipped medical care. These findings are consistent with what another study found [27], although its sample size was much smaller. Some researchers have argued that the US healthcare system is expensive, complicated, and dysfunctional [39]. Therefore, it is unsurprising that benefits and coverage, cost of care, and the process of seeking care are international students’ top concerns regarding healthcare.

In brief, with the median survey score of 52.94 on the test of understanding health insurance vocabulary and 73% potentially feeling confused about their health insurance, the survey participants’ health insurance literacy is not ideal, and obviously, most of them do not have an excellent capacity to explore the US healthcare system. They are not alone because several studies have shown that many Americans have the same issues, as discussed in the introduction. Given the significant contribution of international students, the university where we conducted our study should develop robust initiatives to aid them in understanding their health insurance better.

Orientation is crucial for new international students because it allows them to better understand life in the United States. One student surveyed in a past study pointed out the lack of information sessions on health insurance in the orientation [29]. Not having an introduction to health insurance in the orientation will not equip new international students with the basic skills they need to explore a new healthcare system before officially starting their academic journey. Therefore, the university where we conducted our study should contain a session of reasonable length about health insurance, preferably with a representative from the insurance company, to give international students a thorough introduction to health insurance. Senior peers with similar cultural backgrounds should also be invited to the session to share their experiences using health insurance and accessing medical care.

The student health center is often the first resort when college students need medical care. The staff at the university’s student health center where we conducted the study should receive cultural training periodically to ensure they understand international students’ challenges when seeking medical care. Such an activity will help them know that the assistance this group needs may differ from that of domestic students, and international students may require additional attention during their visits. In addition, the mechanism design of managed care may be new and complex for many international students. Therefore, the center should offer pamphlets in different languages explaining the core health insurance terms in the managed care insurance system. Moreover, the student health center’s website should explain what process can be followed when students need healthcare and how students can gain access when the center is closed.

The university should also create online portals or apps that give their international students easy access to health insurance information and resources. These platforms may include FAQs, instructional videos, and step-by-step guides in multiple languages to help students better understand their coverage and benefits. In addition, collaborating with the international student services office can help ensure that health insurance literacy is adequately addressed in pre-arrival communications and throughout students’ academic journeys. Also, partnering with international student organizations can enhance the dissemination of health insurance information and encourage peer-to-peer knowledge sharing. Moreover, sending periodic reminders and updates about health insurance coverage and policy changes can help international students stay informed and avoid lapses in coverage. Last but possibly the most important, the university should regularly assess the effectiveness of its interventions through surveys, focus groups, or feedback forms. This evaluation helps identify areas for improvement and inform future strategies.

### Limitations

This study has several limitations. First, the imperfect response rate (approximately 20%) of our survey could indicate the existence of nonresponse bias, which is almost impossible to eliminate for any survey. Second, our data are cross-sectional; therefore, causal inference is impossible. Third, questions related to some critical determinants of international students’ health insurance literacy level, such as their English proficiency, are not included in the survey because of concerns about privacy and the time required to complete it. Fourth, no standardized instrument for testing knowledge of health insurance vocabulary is available at this point, so the tool used in this study did not undergo a robust validation process, although it is easy to use. Fourth, this study used convenience sampling, so our sample may not fully represent the studied population. Finally, this study surveyed only international students at one university in the Midwest. Therefore, its results do not help us understand the role of differences in cultural diversity and higher education between regions in shaping international students’ understanding of health insurance. However, the results will guide the design of future related research studies.

### Conclusion

Health insurance literacy is essential for everyone in the United States, especially individuals from other countries. Lack of understanding of health insurance potentially hinders medical care use and impacts people’s health. This study shows that most international students we surveyed, like many Americans, lack health insurance literacy, often feel confused about their health insurance, and do not know how to start their care-seeking process. The literature has indicated that international students are potentially vulnerable because of their complex challenges. Given the significant contribution of international students, the university where we conducted our study should implement appropriate initiatives to help their international students improve their health insurance literacy and access to medical care, especially for newcomers with significantly different cultural backgrounds. As a result, their learning experience will be more pleasant, and their well-being and health will improve.

As indicated in the literature, international students at several universities in the United States also do not have a reasonable understanding of the health insurance system. Presumably, international students at many other colleges have the same challenges, too. Given that the number of international students present at a university makes a significant contribution to the “personality” of that institution and its financial well-being [40], each US university should consider implementing what we suggested in the discussion session or routinely check the effectiveness of their existing initiatives, if any, in helping their international students understand the health insurance system.
