# Supplementary material for: Health Insurance Literacy and Medical Care Avoidance Among International Students: A Case Study
Source: Int J Public Health. 2023 Oct 6;68:1605788. doi: 10.3389/ijph.2023.1605788 (PMC10588627; doi:10.3389/ijph.2023.1605788)
Supplement: Supplementary file 1 [file DataSheet1.docx]

**SUPPLEMENTARY TABLE S1 |** Self-rated understanding of health insurance vocabulary: Before and after completing the questionnaire ($N$ = 143). United States, Midwest 4 February 2022.

|  |  | After | | | | | Total |
| --- | --- | --- | --- | --- | --- | --- | --- |
|  |  | Extremely Well | Very well | Moderately well | Slightly well | Not well at all |  |
| Before | Extremely well | 2 (1.40) | 3 (2.10) | 1 (0.70) | 1 (0.70) | 0 (0.00) | 7 (4.90) |
|  | Very well | 1 (0.70) | 6 (4.20) | 10 (6.99) | 4 (2.80) | 3 (2.10) | 24 (16.78) |
|  | Moderately well | 1 (0.70) | 3 (2.10) | 22 (15.38) | 21 (14.69) | 13 (9.09) | 60 (41.96) |
|  | Slightly well | 1 (0.70) | 0 (0.00) | 4 (2.80) | 14 (9.79) | 16 (11.19) | 35 (24.48) |
|  | Not well at all | 0 (0.00) | 0 (0.00) | 2 (1.40) | 3 (2.10) | 12 (8.39) | 17 (11.89) |
|  | Total | 5 (3.50) | 12 (8.39) | 39 (27.27) | 43 (30.07) | 44 (30.77) | 143 (100) |

**SUPPLEMENTARY TABLE S2 |** Themes and codes in the comments on the experience of ever being confused about using health insurance or picking a health insurance plan (*n* = 87). United States, Midwest 4 February 2022.

| **Themes/**codes | **Example** | ***n* (%)** |
| --- | --- | --- |
| **Benefits and Coverage** |  |  |
| Uncertain of benefits and services included in the plan | - Sometimes, I do not exactly get to understand how much is covered by insurance people and how much I need to put from my pocket - The coverage and plan benefits summary will be confusing | 32 (36.78%) |
| **Cost of care concerns** |  |  |
| Uncertainty surrounding the cost of care | - I don’t know how much money should be paid by myself, so I’ve never used the insurance - It’s rarely straightforward what you have to pay for | 21 (24.14%) |
| **Process** |  |  |
| Uncertainty around the process of obtaining care and using insurance | - I don’t know how to use my health insurance because I am unfamiliar with the process of visiting a doctor in the US, so I don’t know when to use the insurance. | 18 (20.69%) |
| **Terminology** |  |  |
| Uncertainty surrounding the words that are used in their insurance plans | - There are many confusing terminologies - I do not understand copay or clinic-type | 15 (17.24%) |
| **Selecting insurance** |  |  |
| Uncertainty around selecting the right plan for an individual or family | - Confused about choosing the plan which suits the family | 15 (17.24%) |
| **The complexity of US healthcare** |  |  |
| The complexity of the US healthcare system | - It is complicated for no reason - Too complex and complicated | 8 (9.20%) |
| **Lack of exposure** |  |  |
| Have not had exposure to the US health care system | - I had not dealt with this situation yet. My family member always does this. | 4 (4.60%) |

**SUPPLEMENTARY TABLE S3 |** Themes and codes of comments on the experience of ever skipping or delaying medical care due to a lack of understanding of the health insurance plan (*n* = 53). United States, Midwest 4 February 2022.

| **Themes/**codes | **Example** | ***n* (%)** |
| --- | --- | --- |
| **Cost of care concerns** |  |  |
| Uncertainty surrounding the cost of care | - Sometimes, I have headaches, but I do not want to go to the hospital and pay thousands to get them checked | 25 (47.17%) |
| **Benefits and Coverage** |  |  |
| Uncertain of benefits and services included in the plan | - My insurance does not cover much of my medical expenses | 18 (33.96%) |
| **Process of care** |  |  |
| Uncertainty around the process of obtaining care using insurance | - Sometimes, the student health center is closed, and I do not know what to do | 10 (18.87%) |
| **Drug stores** |  |  |
| Avoid care by self-prescribing | - Buy medicine from CVS | 6 (11.32%) |
| **Terminology** |  |  |
| Uncertainty surrounding the words that are used in their insurance plans | - Even though the terms are explained on the health insurance website, they are not easy to understand | 5 (9.43%) |
| **The complexity of US healthcare** |  |  |
| The complexity of the US healthcare system | - It is complicated for no reason - Too complex and complicated | 5 (9.43%) |

**SUPPLEMENTARY FIGURE S1 |** Percentage of respondents who have ever been confused about using health insurance or picking a health insurance plan. United States, Midwest 4 February 2022.

**SUPPLEMENTARY FIGURE S2 |** Percentage of respondents who have ever delayed or avoided medical care due to a lack of understanding of health insurance. United States, Midwest 4 February 2022.
